# Insulin-Like Growth Factor I Produces an Antidepressant-Like Effect and Elicits N-Methyl-D-Aspartate Receptor Independent Long-Term Potentiation of Synaptic Transmission in Medial Prefrontal Cortex and Hippocampus

**DOI:** 10.1093/ijnp/pyv101

**Published:** 2015-09-15

**Authors:** Jeffrey Burgdorf, Xiao-lei Zhang, Elizabeth M. Colechio, Nayereh Ghoreishi-Haack, Amanda Gross, Roger A. Kroes, Patric K. Stanton, Joseph R. Moskal

**Affiliations:** Falk Center for Molecular Therapeutics, Department of Biomedical Engineering, Northwestern University, Evanston, IL (Drs Burgdorf and Moskal); Department of Cell Biology & Anatomy, New York Medical College, Valhalla, NY (Drs Zhang and Stanton); Naurex Inc., Evanston, IL (Dr Colechio, Mrs Ghoreishi-Haack, Dr Gross, Dr Kroes, and Dr Moskal).

**Keywords:** insulin-like growth factor I, depression, long-term potentiation, LTP, N-methyl-d-aspartate receptor, NMDAR, synaptic plasticity

## Abstract

**Background:**

Growth factors play an important role in regulating neurogenesis and synapse formation and may be involved in regulating the antidepressant response to conventional antidepressants. To date, Insulin-like growth factor I (IGFI) is the only growth factor that has shown antidepressant properties in human clinical trials. However, its mechanism of action remains unclear.

**Methods:**

The antidepressant-like effect of a single IV dose of IGFI was determined using a chronic unpredictable stress paradigm in the rat Porsolt, sucrose preference, novelty-induced hypophagia, and ultrasonic vocalization models. The dependence of the medial prefrontal cortex for these effects was determined by direct medial prefrontal cortex injection followed by Porsolt testing as well as IGFI receptor activation in the medial prefrontal cortex following an optimal IV antidepressant-like dose of IGFI. The effect of IGFI on synaptic transmission and long-term potentiation (LTP) of synaptic strength was assessed in the hippocampus and medial prefrontal cortex. The dependence of these effects on IGFI and AMPA receptor activation and protein synthesis were also determined.

**Results:**

IGFI produced a rapid-acting and long-lasting antidepressant-like effect in each of the depression models. These effects were blocked by IGFI and AMPA receptor antagonists, and medial prefrontal cortex was localized. IGFI robustly increased synaptic strength in the hippocampus and medial prefrontal cortex and these effects were IGFI receptor and protein synthesis-dependent but N-methyl-d-aspartate receptor independent. IGFI also robustly facilitated hippocampal metaplasticity 24 hours postdosing.

**Conclusions:**

These data support the conclusion that the antidepressant-like effects of IGFI are mediated by a persistent, LTP-like enhancement of synaptic strength requiring both IGFIR activation and ongoing protein synthesis.

## Introduction

Growth factor activation has been shown to be increased by antidepressant treatment in humans and produce antidepressant-like responses in animal models. Brain derived neurotropic factor, fibroblast growth factor, and insulin-like growth factor I (IGFI) have been shown to be decreased in depressed patients and subsequently elevated by treatment with conventional antidepressants (Duman and Monteggia, 2006; Sen et al., 2008). In addition, continuous (7–14 day) administration of IGFI or brain derived neurotropic factor has been shown to produce an antidepressant-like response in mice (Duman et al., 2009; Schmidt and Duman, 2010).

IGFI has been approved by the FDA for human use in congenital dwarfism (NDA21-839) and may have therapeutic potential for the treatment of depression. IGFI has been reported to have antidepressant and anxiolytic effects in humans (Thompson et al., 1998). In addition, IGFI appears to be unique among growth factors in its ability to readily cross the blood-brain barrier (Reinhardt and Bondy, 1994) and is well tolerated in humans with a minimal side effect profile (NDA21-839). Plasma IGFI levels are positively correlated with positive affective states and negatively correlated with depression scores (Stouthart et al., 2003; Lasaite et al., 2004). In animal studies, IGFI appears to play a functional role in positive affective states associated with rat hedonic 50-kHz ultrasonic vocalizations (USVs) (Burgdorf et al., 2010). IGFI has been implicated in the antidepressant response to SSRIs, and its antidepressant-like effects have been hypothesized to be linked to its ability to facilitate synaptic plasticity (Grunbaum-Novak et al., 2008). Nevertheless, while the molecular biological, biochemical, and pharmacological actions of IGFI have been well characterized (Le Roith, 1997; Fernandez and Torres-Aleman, 2012), the mechanisms underlying either its antidepressant or nootropic effects are not well understood.

 The chronic unpredictable stress (CUS) model has been shown to produce a chronic depressive-like phenotype in rats that is responsive to long-term (3 weeks), but not acute or short-term, antidepressant treatment (Schmidt and Duman, 2007). CUS also causes atrophy of neurons in rodent prefrontal cortex and hippocampus (Radley and Morrison, 2005; Goldwater et al., 2009), effects that could contribute to the decreased volume of these regions reported in brain imaging studies of patients with major depressive disorder (Drevets et al., 2008; Cole et al., 2011).

The experiments reported here were designed to determine if systemically administered IGFI produces an antidepressant-like effect in a rat model of chronic stress-induced depressive-like behavior and to identify underlying neurophysiological mechanisms of the actions of IGFI.

## Methods and Materials

### Animals

Adult, male, 2- to 3-month-old Sprague-Dawley rats purchased from Harlan were used. Rats were housed in Lucite cages with aspen wood chip bedding, maintained on a 12:12 light:dark cycle (lights on at 5:00 am), and given ad libitum access to Purina lab chow and tap water throughout the study. All experiments were approved by the Northwestern University or New York Medical College Animal Care and Use Committees.

### Drugs

IGFI (molecular weight 7655) was purchased from Shenandoah Biotechnology, and the IGFI antagonist JB1 (molecular weight 1249.5), the protein synthesis inhibitor anisomycin, and the NMDA receptor antagonist D-AP5 were purchased from Sigma. The AMPA/Kainate receptor antagonist 2,3-Dioxo-6-nitro-1,2,3,4 tetrahydrobenzo[f]quinoxaline-7-sulfonamide (NBQX) disodium salt was purchased from Abcam. All drugs were administered *in vivo* in sterile saline vehicle at a volume of 1mL/kg and *in vitro* in artificial cerebrospinal fluid (aCSF).

### Porsolt Test

Testing was conducted exactly as described in Burgdorf et al. (2013). Animals were placed in a 46-cm-tall × 20-cm-diameter clear glass tube filled to 30cm with tap water (23±1°C) for 15 minutes on the first day (habituation) and 5 minutes on the subsequent test days (1 hour, 24 hours, 1 week, 2 weeks postdosing). Water was changed after every other animal. Animals were videotaped, and floating time was defined as the minimal amount of effort required to keep the animal’s head above water. Experiments were conducted in a blind manner and scored offline by an experimenter with high inter-rater reliability (Pearson’s r > .9).

#### JB1 Studies

The day after Porsolt habituation, nonchronically stressed rats were dosed with IGFI (0.1mg/kg IV), the IGFIR antagonist JB1 (0.5mg/kg IV), coadministration of both IGFI and JB1, or sterile saline vehicle (1mL/kg IV). All animals received a single 5-minute Porsolt test session 1 hour postdosing. n=8/group.

#### Anisomycin Studies

The day after Porsolt habituation, nonchronically stressed rats were dosed with anisomycin (100mg/kg IP) or sterile saline vehicle (1mL/kg IP) 30 minutes before IGFI (0.1mg/kg IV) or sterile saline vehicle (1mL/kg IV) dosing. All animals received a single 5-minute Porsolt test session 1 hour post IV dosing. n = 6–9/group.

#### NBQX Studies

The day after Porsolt habituation, nonchronically stressed rats were dosed with NBQX (10mg/kg IP) or sterile saline vehicle (1mL/kg IP) 70 minutes before testing. Animals were these dosed with IGFI (0.1mg/kg IV) or sterile saline vehicle (1mL/kg IV) 60 minutes before testing. All animals received a single 5-minute Porsolt test session. n = 8/group.

### Open Field Test

Open-field testing was performed as previously described (Burgdorf et al., 2013). Testing consisted of placing an animal in a 40×40×20cm high, opaque Plexiglas open-field cage divided into 9 equally sized 13.3-×13.3-cm sections under red lighting for 10 minutes. Between animals, feces and urine were removed from the apparatus. Animals were videotaped, and line crosses were scored offline by blind experimenters with high inter-rater reliability (Pearson’s r > 0.9).

As described above, IGFI (0.1mg/kg IV), JB1 (0.5mg/kg IV), or sterile saline vehicle (1mL/kg IV) was administered 60 minutes before testing. Anisomycin (100mg/kg IP) or sterile saline vehicle (1mL/kg IP) was administered 90 minutes before testing. NBQX (10mg/kg IP) or sterile saline vehicle (1mL/kg IP) was administered 70 minutes before testing. n = 6/group.

### Microinjection Surgery

Unilateral 22-gauge guide cannulae (Plastic Products) were stereotaxically implanted into the infralimbic/prelimbic cortex regions of the medial prefrontal cortex (MPFC; 2.7mm anterior, ±0.5mm lateral, 3.0mm ventral to bregma; flat brain) under isoflurane anesthesia as previously described (Burgdorf et al., 2013). We have previously shown that surgical doses of isoflurane (2–5% for 20 minutes) does not, by itself, induce an antidepressant-like effect in rats (Burgdorf et al., 2013). All animals were allowed 1 week to recover from surgery before the start of testing. After the completion of behavioral testing, histology was conducted, and all cannulae tips were located within the infralimbic or prelimbic cortex 2.2 to 3.2mm anterior to bregma.

### CUS Procedure

Rats were exposed to a CUS protocol previously shown to elicit depression-like symptoms in rats (Li et al., 2011). Animals received 21 days of CUS before dosing and continued to receive CUS until they were sacrificed 1 day after the last behavioral test (total of 37 days of CUS). A total of 9 different CUS stressors were used (2 stressors/d). The stressors (days) included rotation on a shaker for 1 hour (3, 9, 13, 19, 24, 28, 33, 37), placement in a 4°C ambient for 1 hour (1, 5, 12, 14, 18, 22, 26, 30, 36), lights off for 3 hours from 10:00 am to 1:00 pm (2, 10, 17, 23, 31, 34, 37), lights on overnight (1, 5, 8, 13, 16, 22, 32, 34), strobe light overnight (3, 6, 9, 14, 17, 20, 23, 28, 31, 33), 45° tilted cages overnight (4, 7, 11, 15, 18, 21, 25, 29, 35), food and water deprivation overnight (2, 6, 10, 15, 19, 26, 27, 30), crowded housing overnight (4, 7, 11, 16, 21, 25, 29, 35), and isolation housing overnight (8, 12, 20, 24, 27, 32, 36). Animals in the No CUS control group (n=9) were weighed every 4 days and received behavioral testing without additional stressors. Animals in the CUS groups received a single optimal dose of IGFI (0.1mg/kg IV; n=10) or sterile saline vehicle (n=10).

### Sucrose Preference Test

Testing was conducted exactly as described in Li et al. (2011), and testing occurred 3 days postdosing. Rats were exposed to a palatable sucrose solution (1%; Sigma) for 48 hours, followed by 4 hours of water deprivation and a 1-hour exposure to 2 identical bottles, one filled with sucrose solution and the other with tap water. Sucrose preference was defined as the ratio of the volume of sucrose vs total volume of sucrose and water consumed during the 1-hour test.

### Novelty Induced Hypophagia (NIH) Test

Testing was conducted as described in Burgdorf et al. (2013), and testing occurred 2 days postdosing. Animals were food deprived on the night before testing, and lab chow was placed into the center chamber of the open field (40×40×20cm) for 10 minutes under dim-red lighting. Between animals, feces and urine were removed from the apparatus. Immediately after NIH testing, the latency to eat in the animal’s home cage was determined as a control. Animals were videotaped, and latency (seconds) for the animal to take the first bite of food, as well as locomotor activity (line crosses) was scored offline by an experimenter blinded to the treatment condition. Home cage food intake was also measured during the first hour and first 24 hours after NIH testing.

### USV Test

Heterospecific rough-and-tumble play was conducted exactly as previously described (Burgdorf et al., 2013), and testing occurred 3 hours and 2 weeks postdosing. Heterospecific rough-and-tumble play stimulation was administered by the experimenter’s right hand. The experimenter was blind to the treatment condition of the animals. Animals received 3 minutes of heterospecific rough-and-tumble play consisting of alternating 15-second blocks of heterospecific play and 15 seconds of no stimulation. High-frequency USVs were recorded and analyzed by sonogram in a blind manner as previously described (Burgdorf et al., 2011b). Animals were not habituated to play stimulation before dosing and testing. Using this paradigm, we have shown that the increase in 50-kHz USVs that occur across trial blocks reflects positive emotional learning (Burgdorf et al., 2011b).

### Phosphotyrosine 1311 IGFI Receptor Protein Quantification

One hour postdosing with IGFI (0.1mg/kg IV), JB1 (0.5mg/kg IV), IGFI + JB1, or sterile saline vehicle (1mL/kg IV), rat brains were rapidly removed following decapitation (approximately 60 seconds), and the MPFC dissected on an ice-cold platform and stored at -80°C until assay. Tissue was sonicated in ice-cold RIPA buffer (9806, Cell Signaling) supplemented with protease and phosphatase inhibitors (Sigma), centrifuged at 25000 *g* for 10 minutes at 4°C, supernatant removed, and protein content determined by the BCA assay. Phospho-tyrosine 1311 IGFIR protein levels were quantified by ELISA (7302, Cell Signaling), and all samples were within the linear range of the assay.

### Sulfo-NHS-SS-Biotinylation of Cell Surface Protein

Methods were conducted as previously described (Burgdorf et al., 2013). In brief, 24 hours postdosing with IGFI (0.1mg/kg, IV) or sterile saline vehicle (1mL/kg IV), rat brains were rapidly removed following decapitation (approximately 60 seconds), the MPFC and hippocampus were dissected on an ice-cold platform, and MPFC and hippocampal sections (approximately 300 micron) were washed then incubated in ice-cold sulfo-NHS-SS-Biotin (1mg/mL) for at least 30 minutes. Tissue was then frozen on dry ice and stored at −80°C until assay. Tissue extracted in ice-cold RIPA buffer, and protein (3mg) was precipitated with NeutrAvidin Ultralink resin (Pierce) overnight at 4°C with agitation. Protein samples were separated by SDS-polyacrylamide gel electrophoresis and transferred onto PVDF membranes. Membranes were incubated with GluR1 antibody (MAB2263, 1:500, Millipore), NR2B antibody (4207S, 1:500, Cell Signaling) in 3% NMDF TBS overnight at 4°C, followed by a 1-hour incubation at 25°C with a HRP-conjugated secondary antibody (1:2000–1:5000, Santa Cruz Biotechnology). Immunoreactive bands were visualized by enhanced chemiluminescence (Immun-Star HRP, Bio-Rad) and exposed to film (BioMax, Kodak) for appropriate times. Membranes were reprobed with β-actin (5125, 1:1000, Cell Signaling) for normalization. All images were within the linear range of the film and were quantified by ImageJ software (NIH).

### Slice Electrophysiology

In vitro slice electrophysiology experiments were conducted as described previously (Burgdorf et al., 2013). Animals were anesthetized with isoflurane and decapitated. Brains were rapidly removed and submerged in ice-cold aCSF (2–4°C), which contained (in mM): 124 NaCl, 2.5 KCl, 1.5 MgSO_4_, 2.5 CaCl_2_, 1.25 NaH_2_PO_4_, 26 NaHCO_3_, 10 glucose at pH 7.4, gassed continuously with 95% O_2_/5% CO_2_. Brains were hemisected and individual hemispheres glued using cyanoacrylate adhesive onto a stage immersed in ice-cold aCSF gassed continuously with 95% O_2_/5% CO_2_ during slicing. The 400-micron-thick coronal hippocampal or MPFC slices containing both the prelimbic and infralimbic regions of the MPFC that are targets (via the fornix) of the hippocampal-MPFC pathway (Parent et al., 2010) were cut using a vibratome (Leica VT1200S) and transferred to an interface holding chamber for incubation at room temperature for a minimum of 1 hour before transferring to a Haas-style interface recording chamber continuously perfused at 3mL/min with oxygenated aCSF at 32±0.5°C.

Low-resistance recording electrodes were made from thin-walled borosilicate glass (1–2 MΩ after filling with aCSF) and inserted into the apical dendritic region of the Schaffer collateral termination field in stratum radiatum of field CA1 region to record field excitatory postsynaptic potentials (fEPSPs). A bipolar stainless-steel stimulating electrode (**In Vivo**) was placed on Schaffer collateral-commissural fibers in CA3 stratum radiatum or inserted into layer III/IV of the prelimbic region of MPFC slices and constant current stimulus intensity adjusted to evoke approximately half-maximal fEPSPs once each 30 seconds (50–100 pA; 100 μs duration). fEPSP slope was measured by linear interpolation from 20 to 80% of maximum negative deflection and slopes confirmed to be stable to within ±10% for at least 15 minutes before commencing an experiment. Signals were recorded using a differential AC amplifier (A-M systems Model 1700) and digitized with an A/D board from DataWave Technologies. Electrophysiological data collection was operated, controlled, and analyzed with DataWave SicWorks (v7.2 SP1, Loveland, CO) running on a PC.

### Whole-Cell Patch-Clamp Intracellular Recording

Whole-cell patch-clamp recordings from hippocampal CA1 pyramidal neurons were performed as described previously (Burgdorf et al., 2013) using a MultiClamp 700B amplifier (Molecular Devices, Union City, CA). Patch pipette resistance ranged from 6 to 6.5 MΩ when filled with intracellular solution that contained (in mM): 135 CsMeSO_2_, 8 NaCl, 10 HEPES, 0.2 EGTA, 2 Mg-ATP, 0.3 Na-GTP, and 1 N-(2,6-dimethylphenylcarbamoylthyl)-triethylammonium bromide, 275 mOsm, pH 7.25 adjusted with Cs(OH)_2_. Neurons were visualized by infrared imaging and patched using a 60×/1.1-nA water-immersion objective mounted to a Zeiss microscope (Axioskop 2 Fs plus). After whole-cell voltage clamp configuration was established, access resistance was carefully monitored, and only cells with stable access resistance (<5% change) were included for analyses. Excitatory postsynaptic currents were filtered at 3kHz and digitized at 10kHz with a Digidata 1322A controlled by a Clampex (v9.2) (Molecular Devices, Union City, CA). A bipolar tungsten stimulating electrode (FHC, Bowdoin, ME) was placed in the mPFC deep white matter input, at least 200 µm from the patched neuron, and stimulus pulses (80-µS duration) were delivered at 15-second intervals. Neurons were voltage clamped at -70 mV to record excitatory postsynaptic currents to assess input-output relations and paired-pulse facilitation.

All external recording pipette solutions were made with deionized distilled water (resistance >18 MΩ cm^−2^; Milli-Q system; Millipore). Chemicals for making extra- and intracellular solutions were purchased from Sigma or Fluka. After electrophysiological data were analyzed initially with DataWave SciWorks, they were further processed and presented with Origin 6.1 (Microcal Software) and CorelDraw 10.0 (Corel) programs.

### Acute Slice Drug Application

Slices were transferred to an interface recording chamber and continuously perfused at 3mL/min with oxygenated aCSF at 32°C, and half-maximal fEPSPs, once each 30 seconds (50–100 pA; 100 µS duration), were recorded. fEPSP slopes were conﬁrmed to be stable to within ±10% for at least 10 minutes before commencing an experiment. Slices were pretreated with the IGFIR antagonist JB1 (1 µM; purple circles), the protein synthesis inhibitor anisomycin (10 µM), the NMDA receptor antagonist D-AP5 (25 µM; green circles), or aCSF alone 10 minutes before the addition of IGFI (200nM) at a 3-mL/min flow rate. For statistical analysis, normalized excitatory postsynaptic potentials 53 to 56 minutes after the addition of IGFI were determined.

### *In Vivo* IGFI Effects on LTP

Twenty-four hours after dosing with IGFI (0.1mg/kg IV) or sterile saline vehicle (1mL/kg IV) hippocampal slices were prepared. LTP was induced by stimulation of Schaffer collateral axons with 2 high-frequency theta burst stimulus trains of 10×100 Hz/5 pulse bursts each, applied at an inter-burst interval of 200 millisecnods. Each train was 2 seonds in duration, and trains were applied 3 minutes apart. The tentanizating stimuli with the same stimulus pattern were repeated every 15 minutes an additional 2 times as shown in Figure 5. For statistical analysis, LTP was measured 15 minutes after the first and second tetanus and 30 minutes after the third tetanus.

### Statistical Analysis

Behavioral, biochemical, and electrophysiological data were analyzed by ANOVA, followed by Fisher’s PLSD posthoc test (Statview). The level of statistical significance was preset to *P* < .05.

## Results

### A Single IV Dose of IGFI Produces an IGFIR-Dependent Antidepressant-Like Effect in the Porsolt Test following IV or Intra-MPFC Injection

**Figure 1.**
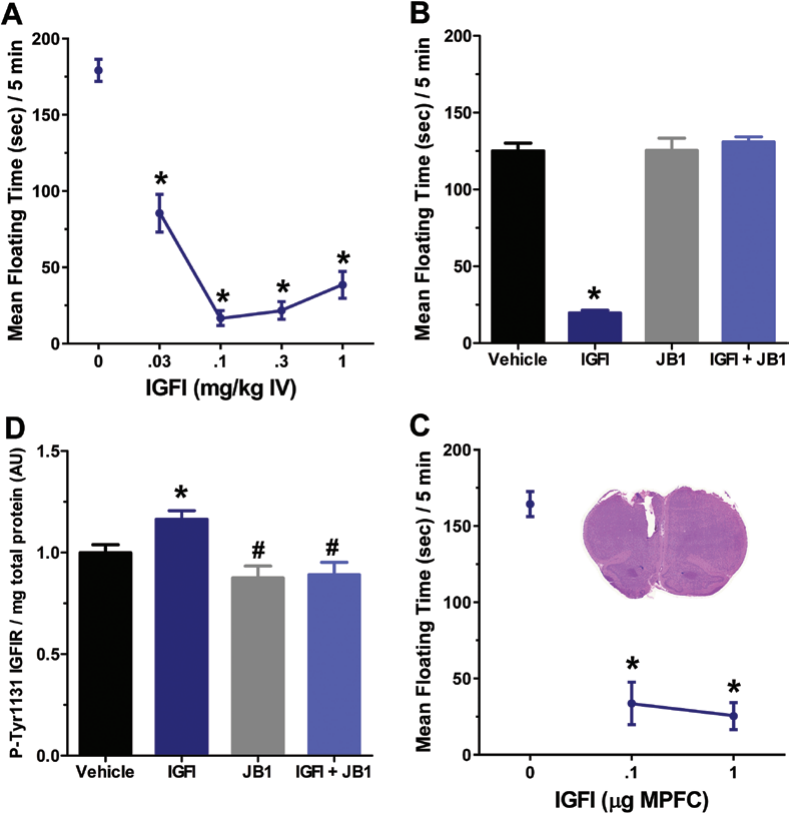
Insulin-like growth factor I (IGFI) produces an IGFIR dependent antidepressant-like response following IV and intra-MPFC injection in rats, and activates the IGFIR in the medial prefrontal cortex (MPFC) following IV injection. Mean±SEM floating time in non-chronic unpredictable stress (CUS)–exposed rats treated with (A) a single IV injection of IGFI (0.03 to 1mg/kg), saline vehicle (1mL/kg), and tested 1 hour postdosing, (B) a single IV injection of IGFI (0.1mg/kg; 131 nMol/kg), the IGFIR antagonist JB1 (0.5mg/kg; 4002 nMol/kg), or coadministration of IGFI and JB1. (C) Mean±SEM phospho-tyrosine1311 IGFIR protein levels in the MPFC of non-CUS-treated rats 1 hour after a single injection of IGFI (0.1mg/kg IV), JB1 (0.5mg/kg IV), IGFI + JB1, or saline vehicle (1mL/kg). (D) A single unilateral MPFC injection of IGFI (0.1 to 1 µg) or saline vehicle (0.5 µL) and tested 15 minutes postdosing. (D, inset) A representative H&E-stained section depicting MPFC cannulae placement. Additionally, IGFI (0.1mg/kg IV, or 1 µg intra-MPFC) did not alter locomotor activity in the open field 1 hour postdosing (IV) or 15 minutes (MPFC) postdosing (results section). *P<.05 Fisher’s PLSD posthoc test vs vehicle. n = 6 to 13/group.

As shown in Figure 1A, IGFI (0.03 to 1mg/kg IV) produced an antidepressant-like effect in the Porsolt test 1 hour postdosing [*F*(4, 35) = 66.8, *P*<.05; Fisher’s PLSD posthoc test all IGFI doses vs vehicle], with the 0.1-mg/kg IV dose being the lowest maximally effective dose [Fishers PLSD posthoc test 0.1 vs 0.03, *P*<.05; 0.1 vs all other IGFI doses, *P*<.05]. As shown in Figure 1B, the antidepressant-like effect of IGFI (0.1mg/kg IV) was blocked by coadministration of a silent dose of the IGFIR antagonist JB1 (0.5mg/kg IV) 1 hour postdosing [*F*(3, 20) = 111.5, *P*<.05; Fisher’s PLSD posthoc test all IGFI alone vs all other groups, *P*<.05; JB1 vs vehicle, *P*<.05]. As shown in Figure 1C, a single injection of IGFI (0.1mg/kg IV) increased protein levels of phospho-IGFI receptor β (tyrosine 1131), and this effect was blocked by coadministration of the IGFIR antagonist JB1 [*F*(3, 34) = 7.8, *P*<.05, Fisher’s PLSD posthoc test IGFI vs vehicle, IGFI vs JB1, IGFI vs JB1 + IGFI, *P*<.05]. As shown in Figure 1D, MPFC injections of IGFI (0.1–1 µg, unilaterally) also produced an antidepressant-like effect in the Porsolt test 1 hour postdosing as well as after retesting 24 hours postdosing [*F*(4, 35)=47.8, *P*<.05; Fisher’s PLSD posthoc test all IGFI doses vs respective vehicle, *P*<.05].

In addition, IGFI (0.1mg/kg IV, 1 hour postdosing; 1 µg intra-MPFC, 20 minutes postdosing) did not alter locomotor activity in the open field as measured by line crosses compared with vehicle [IV dosing: F(1, 10) = 0.3, *P*<.05; line crosses/10 minutes (mean±SEM), vehicle (220.0±20.2), IGFI (202.0±28.9); MPFC dosing: F(1, 6)=0.3, *P*<.05; line crosses/10 minutes (mean±SEM), vehicle (207.8±32.7), IGFI (184.0±25.9)]. However, IGFI did increase time spent in the center compartment of the open field following both routes of administration [IV dosing: F(1, 10) = 10.3, *P*<.05; center time (sec)/10 minutes (mean±SEM), vehicle (16.8±4.7), IGFI (47.4±8.3); MPFC dosing: F(1, 6) = 34.6, *P*<.05; center time (seconds)/10 minutes (mean±SEM), vehicle (14.2±3.9), IGFI (87.8±11.9)].

### A Single IV Dose of IGFI Produces a Long-Lasting Antidepressant-Like Effect in the Porsolt, Sucrose Preference, and NIH Tests in Rats Exposed to CUS

**Figure 2.**
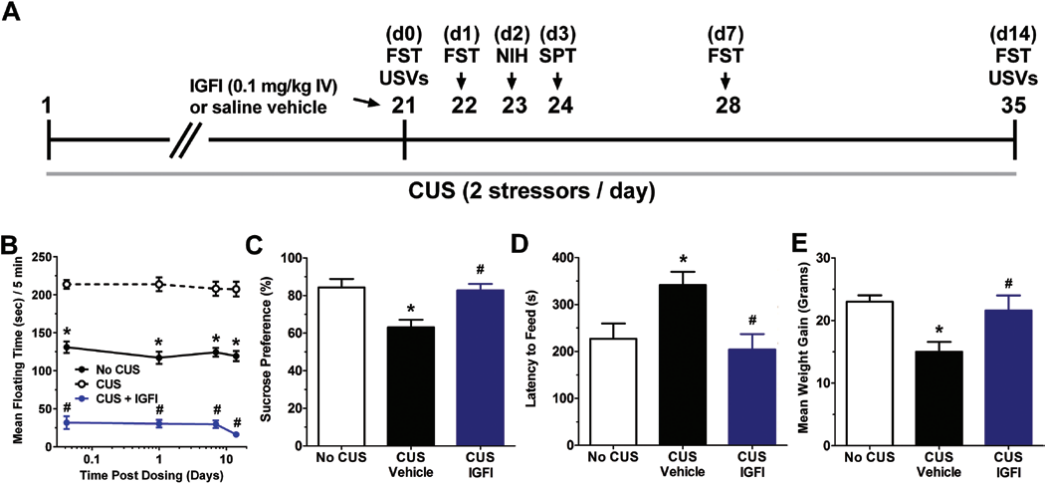
Insulin-like growth factor I (IGFI) produces an antidepressant-like effect in a chronic unpredictable stress (CUS) paradigm. (A) Schematic demonstrating the timeline for CUS exposure, drug administration, and behavioral testing. Numbers in parentheses represent days after drug administration. Rats were exposed to CUS and administered IGFI (0.1mg/kg IV) on day 21. (B) Mean±SEM floating time in the Porsolt forced swim test (FST) in CUS-treated rats treated with a single dose of IGFI (0.1mg/kg IV; n = 10), saline vehicle (n = 10), or no CUS-exposed control rats (n = 9) tested 1 hour, 1 day, 1 week, and 2 weeks postdosing. Mean±SEM. (C) Sucrose preference in the sucrose preference test (SPT), 4 days postdosing, (D) latency to feed in the NIH test (3 days postdosing), (E) body weight (2 weeks postdosing). *P<.05 Fisher’s PLSD posthoc test vs no CUS group, #*P*<.05 Fisher’s PLSD posthoc test vs CUS vehicle group. n = 9 to 10/group.

As shown in Figure 2A, IGFI (0.1mg/kg, IV) in rats exposed to CUS significantly reduced floating time in the Porsolt test (1 hour, 1 day, 1 week, and 2 weeks postdosing) compared with CUS-treated vehicle rats, and CUS vehicle rats showed increased floating times compared with no CUS-treated rats [*F*(2, 113) = 667.6, *P*<.05; Fisher’s PLSD posthoc test IGFI vs vehicle, or vehicle vs no CUS control, *P*<.05]. As shown in Figure 2B, IGFI increased sucrose preference scores in CUS-treated rats (4 days postdosing) compared with CUS-treated vehicle rats, while CUS vehicle-treated rats showed significantly decreased sucrose preference scores compared with no CUS-treated rats [*F*(2, 26)=9.1, *P*<.05; Fisher’s PLSD posthoc test IGFI vs vehicle, vehicle vs no CUS control, *P*<.05]. As shown in Figure 2C, IGFI also significantly decreased feeding latency in a novel environment in the NIH test (2 days postdosing) compared with CUS-treated vehicle rats, while CUS vehicle rats showed longer feeding latencies compared with no CUS-treated rats [*F*(2, 26) = 5.7, *P*<.05; Fisher’s PLSD posthoc test IGFI vs vehicle, vehicle vs no CUS control, *P*<.05], but no changes were seen in latency to eat in the homecage [*F*(2, 26) = 0.4, *P*<.05; data not shown] or grams of food consumed in the home cage for 1 hour [*F*(1, 16) = 0.1, *P*<.05; data not shown] or 24 hours after testing [*F*(1, 16)=0.2, *P*<.05; data not shown], or line crosses in the novel environment [*F*(2, 26) = 0.3, *P*<.05; data not shown]. As shown in Figure 2D, IGFI increased weight gain (2 weeks postdosing) compared with CUS-treated vehicle rats, while CUS vehicle rats showed decreased weight gain compared with no CUS-treated rats [*F*(2, 26) = 5.6, *P*<.05; Fisher’s PLSD posthoc test IGFI vs vehicle, vehicle vs no CUS control, *P*<.05].

### IGFI Produces a Long-Lasting Antidepressant-Like Effect in the USV Test and Increased Positive Emotional Learning in Rats Exposed to CUS

**Figure 3.**
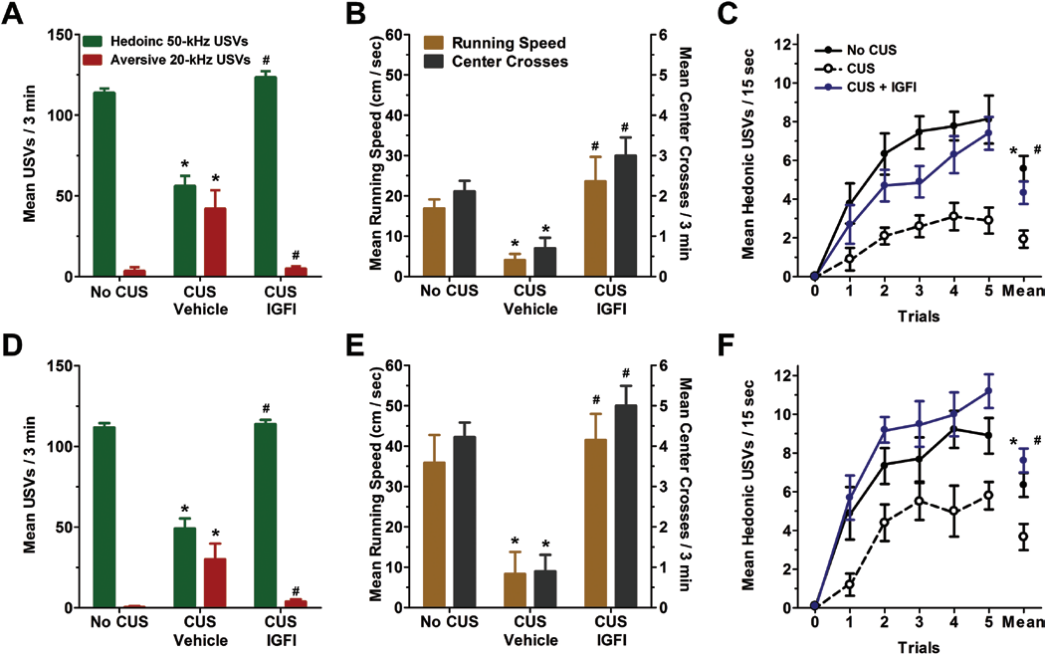
Insulin-like growth factor I (IGFI) decreases aversive ultrasonic vocalizations (USVs) and increases both hedonic USVs and positive emotional learning in a chronic unpredictable stress (CUS) paradigm. Mean±SEM hedonic and aversive USVs in CUS-treated rats treated with a single dose of IGFI (0.1mg/kg IV; n = 10), saline vehicle (n = 10), or no CUS-exposed control rats (n=9) tested 3 hours (A) and 2 weeks (D) postdosing. Mean±SEM running speed to self-administer heterospecific play and center crosses 3 hours (B) and 2 weeks (E) postdosing. Mean±SEM hedonic USVs in response to a conditioned stimuli that predicts heterospecific play 3 hours (C) and 2 weeks (F) postdosing. *P<.05 Fisher’s PLSD posthoc test vs no CUS group, #*P*<.05 Fisher’s PLSD posthoc test vs CUS vehicle group.

As shown in Figure 3A (3 hours postdosing) and 3D (2 weeks postdosing), IGFI in CUS-treated rats increased rates of hedonic USVs and reduced rates of aversive 20-kHz compared with vehicle CUS-treated rats, while CUS vehicle-treated rats showed decreased hedonic USVs and increased aversive USVs compared with no CUS-treated rats [hedonic USVs *F*(2, 26) = 65.1 (3 hours), 61.7 (2 weeks), *P*<.05; aversive USVs *F*(2, 26) = 9.9 (3 hours), 8.9 (2 weeks), *P*<.05 Fisher’s PLSD posthoc test IGFI vs vehicle, vehicle vs no CUS control, *P*<.05]. As shown in Figure 2B (3 hours postdosing) and 2E (2 weeks postdosing), IGFI in CUS-treated rats significantly increased both running speed to self-administer heterospecific play and center crosses compared with vehicle CUS-treated rats, while CUS vehicle rats showed decreased running speed and center crosses compared with no CUS-treated rats [running speed *F*(2, 26)=9.9 (3 hours), 7.2 (2 weeks), *P*<.05; center crosses *F*(2, 26)=9.9 (3 hours), 27.4 (2 weeks), *P*<.05 Fisher’s PLSD posthoc test IGFI vs vehicle, vehicle vs no CUS control, *P*<.05] but did not alter locomotor activity as measured by line crosses [*F*(2, 26)=0.6 (3 hours), 0.7 (2 weeks), *P*<.05; data not shown]. As shown in Figure 3C (3 hours postdosing) and 3F (2 weeks postdosing), IGFI in CUS-treated rats increased rates of hedonic USVs in response to a temporal CS that predicted heterospecific play compared with vehicle-CUS-treated rats, and CUS vehicle rats showed decreased CS elicited hedonic USVs compared with no CUS-treated rats [*F*(2, 26)=10.9 (3 hours), 10.3 (2 weeks), *P*<.05; Fisher’s PLSD posthoc test IGFI vs vehicle, vehicle vs no CUS control, *P*<.05].

### Bath Application of IGFI Facilitates Synaptic Transmission in the Hippocampus and MPFC Requiring IGFIR and Protein Synthesis, but Not NMDA Receptor Activation

**Figure 4.**
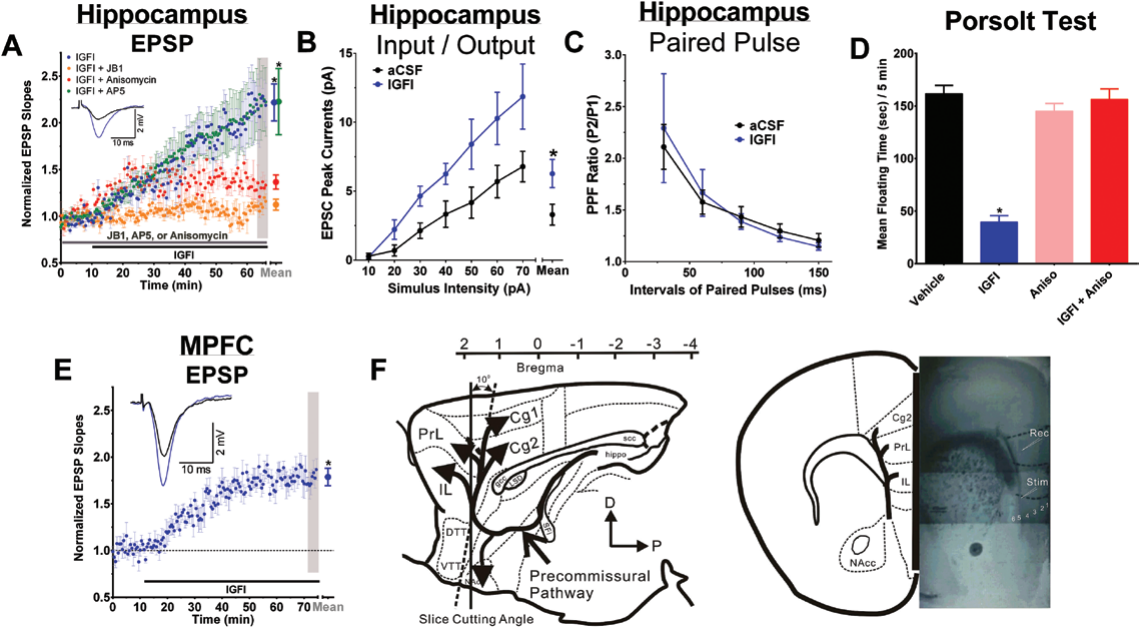
Acute insulin-like growth factor I (IGFI) enhances synaptic strength in hippocampus and medial prefrontal cortex (MPFC) via a postsynaptic process that is protein synthesis dependent, but NMDAR independent, and the antidepressant-like effect of IGFI is also protein synthesis dependent. (A) Evoked excitatory postsynaptic potential (EPSP) slopes in hippocampal slices treated with IGF1 alone (200nM; black bar, blue circles) vs slices treated with IGF1 in the presence of (grey bar) the IGFIR antagonist JB1 (1 µM; purple circles), protein synthesis inhibitor anisomycin (10 µM; red circles), or the NMDA receptor antagonist (25 µM; green circles). (B) Input/output curves and (C) paired-pulse facilitation in hippocampal slices following 50-minute bath application of artificial cerebrospinal fluid (aCSF) or IGFI (200nM). (D) Floating time in the Porsolt test in non-chronic unpredictable stress (CUS)-pretreated animals first dosed with anisomycin (100mg/kg IP) or sterile saline vehicle (IP) 30 minutes before IGFI (0.1mg/kg IV) or saline vehicle (IV) and tested 1 hour after IV dosing. n = 6 to 9 rats/group. (E) EPSP slopes in MPFC slices treated with IGFI (200nM). (F) Schematic showing the orientation and electrode placement in MPFC slices. n = 5 to 12 slices/group (A,B,C,E); n = 6 to 9/ group (D). Mean±SEM. * P < .05 (A) Fisher’s PLSD posthoc test IGFI or IGFI + AP5 vs JB1, IGFI or IGFI + AP5 vs Anisomycin, or (D) IGFI vs all other groups; (B) ANOVA aCSF vs IGFI; or (E) within-subjects *t* test, 2 tailed, compared with baseline.

Figure 4A demonstrates that a 50-minute bath application of IGFI increased the magnitude of excitatory postsynaptic potentials at Schaffer collateral-CA1 synapses, and this effect was blocked by either the IGFIR antagonist JB1 or the protein synthesis inhibitor anisomycin, but not the NMDA receptor antagonist D-AP5 [*F*(3,18) = 5.6, *P*<.05; Fisher’s PLSD posthoc test IGFI or IGFI + AP5 vs all other groups, *P*<.05; IGFI vs IGFI + AP5, *P*<.05]. IGFI significantly shifted the input/output curve to the left, indicating increased EPSP magnitude across the full range of synaptic transmission [*F*(1,13)=5.2, *P*<.05; Figure 4B] but did not alter paired-pulse facilitation [*F*(1,19) = 0.0, *P*<.05] (Figure 4C), indicating a postsynaptic site of action of IGFI.

As shown in Figure 4D, a 50-minute bath application of IGFI also increased the amplitude of EPSPs in the MPFC compared with baseline [*t*(8) = 8.45 *P*<.05, within-subjects *t* test, 2-tailed].

### The Antidepressant-Like Effect of IGFI Is Protein Synthesis Dependent

Figure 4E shows that pretreatment with a silent dose of anisomycin blocked the antidepressant-like effect of IGFI in the Porsolt test [*F*(3,25) = 47.6, *P*<.05; Fisher’s PLSD posthoc test IGFI vs all other groups].

### The AMPA/Kainate Receptor Antagonist NBQX, 24 Hours Postdosing, Occludes the Antidepressant-Like Effect of IGFI in the Porsolt Test

**Figure 5.**
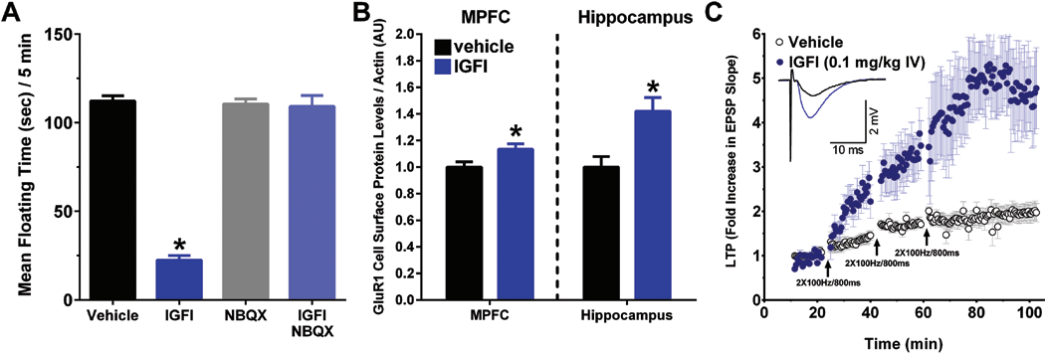
The long-lasting antidepressant-like effects of insulin-like growth factor I (IGFI) are AMPA receptor-dependent and associated with a long-lasting, metaplastic increase in induction threshold and magnitude of LTP. (A) Mean (±SEM) floating time in the Porsolt test in non-chronic unpredictable stress (CUS) -pretreated animals first dosed with 2,3-Dioxo-6-nitro-1,2,3,4 tetrahydrobenzo[f]quinoxaline-7-sulfonamide (NBQX) or saline vehicle (10mg/kg ip; 70 minutes before testing) followed by IGFI or saline vehicle (0.1mg/kg IV; 60 minutes before testing). (B) Mean±SEM cell surface protein levels in the medial prefrontal cortex (MPFC) or hippocampus as measured by Western analyses in rats treated with IGFI (0.1mg/kg, IV) or sterile saline vehicle (1ml/kg IV) 24 hours before sacrifice. MPFC or hippocampus slices were incubated with Sulfo-NHS-SS-Biotin to label surface protein, and biotinylated protein was precipitated with avidin-agarose beads. Protein samples were analyzed by SDS-polyacrylamide gel electrophoresis and transferred to PVDF membranes probed with GluR1- and β-actin-specific antibodies. n=7 to 9 per group. (C) A single *in vivo* dose of IGFI (0.1mg/kg, IV; filled blue circles) in non-CUS-treated rats significantly enhanced the magnitude of LTP compared with saline vehicle-treated controls (open black circles) in hippocampal slices *in vitro* 24 hours postdosing at Schaffer collateral-CA1 synapses after 1, 2, and 3 submaximal high-frequency stimulus trains (2×100 Hz/800ms, arrows, *P*<.05, Fisher’s PLSD posthoc test), n = 4 to 12 slices/group.* *P*<.05 vs vehicle).

As shown in Figure 5A, a silent dose of NBQX administered 10 minutes before dosing with IGFI (0.1mg/kg IV) blocked the antidepressant-like effect of IGFI [*F*(3,32)=125.6, *P*<.05; Fisher’s PLSD posthoc test IGFI only vs all other groups, *P*<.05]. In addition, NBQX, administered 24 hours after dosing with IGFI (0.1mg/kg IV) blocked the antidepressant-like effect of IGFI [*F*(3,28) = 98.7, *P*<.05; Fisher’s PLSD posthoc test IGFI only vs all other groups, *P*<.05, data not shown].

### IGFI, JB1, Anisomycin, and NBQX Did Not Alter Locomotor Activity in Open Field Tests

Neither IGFI (mean±SEM; 46.5±3.0) nor JB1 (mean±SEM; 51.0±5.9) altered line crosses in the open field compared with vehicle (mean±SEM; 45.5±3.0) -treated rats [*F*(2,15) = 0.5, *P*<.05]. Anisomycin (mean±SEM; 32.7±2.1) did not alter line crosses in the open field compared with vehicle (mean±SEM; 36.3±5.2) -treated rats [*F*(1,10) = 0.4, *P*<.05]. NBQX (mean±SEM; 66.3±10.0) did not alter line crosses in the open field compared with vehicle (mean±SEM; 58.3±3.6) -treated rats [*F*(1,10) = 0.6, *P*<.05].

### IGFI Increases Cells Surface GluR1 Protein Levels in the MPFC and Hippocampus 24 Hours Postdosing

Figure 5B shows that IGFI (0.1mg/kg, IV) increased cell surface protein levels of GluR1 in the MPFC as normalized to β-actin protein levels [F(1,14) = 5.5, *P*<.05] and hippocampus [F(1,15) = 10.8, *P*<.05] 24hours postdosing as compared with vehicle.

### IGFI Persistently Enhanced the Induction of Long-Term Potentiation of Synaptic Transmission in the Hippocampus

As shown in Figure 5C, IGFI (0.1mg/kg IV) persistently enhanced ex vivo Schaffer collateral LTP 24 hours postdosing as compared with vehicle 15 minutes after the first tetanus [*F*(1,12) = 17.8, *P*<.05], 15 minutes after the second tetanus [*F*(1,12) = 20.8, *P*<.05], as well as 40 minutes after the third tetanus [*F*(1,12) = 13.3, *P*<.05], indicating that IFGI persistently lowered the threshold for induction of LTP, and raised the ceiling magnitude of LTP, for up to 24 hours after administration.

## Discussion

Growth factors play important roles in regulating neurogenesis and synapse formation and may be involved in regulating the response to conventional antidepressants. To date, IGFI is the only growth factor that has exhibited antidepressant properties in human clinical trials, yet its mechanism of action remains unclear. Here, we report that a single IV injection of IGFI produced a rapid-acting and long-lasting antidepressant-like effect in rats and that these changes required protein synthesis-dependent long-term changes in LTP-like synaptic plasticity. In addition, the rapid-acting (within 1 hour) and long-lasting (2 weeks) antidepressant effects of IGFI reported here suggest that bolus dosing could be used at weekly intervals, possibly decreasing the potential impact of side effects of IGFI such as hypoglycemia.

The CUS model displays both positive and negative symptoms of depression (Li et al., 2011) and can be used to study both rapid-acting and long-lasting antidepressant-like effects. In these studies, the CUS model was used to create chronically depressed rats that were then tested in a variety of behavioral models typically used to screen candidate drugs for their antidepressant properties: the Porsolt, NIH, sucrose preference, and rat USV tests. These particular tests were chosen for the following reasons: The Porsolt test has been shown to have high predictive validity and can both detect the acute antidepressant-like effects of ketamine and requires chronic administration of SSRIs to show efficacy (Cryan et al., 2002; Li et al., 2010; Burgdorf et al., 2013). In a similar manner, we used a version of the NIH test that has previously been shown to detect the acute antidepressant-like effect of ketamine (Li et al., 2010), but require chronic administration of fluoxetine to detect an antidepressant-like effect (Dulawa and Hen, 2005; Burgdorf et al., 2013). The sucrose preference test captures the anhedonic symptoms of depression. Finally, the USV test can simultaneously measure both positive (hedonic 50-kHz USVs) and negative (aversive 20-kHz USVs) emotional states relevant to depression (Burgdorf et al., 2011a).

In this study, IGFI persistently enhanced synaptic strength and activity-dependent long-term synaptic plasticity via a mechanism that required both IGFIR and AMPAR activity and protein synthesis and is likely to be due to postsynaptically sites of induction and expression. NMDAR-dependent forms of long-term potentiation are triggered by increases in postsynaptic [Ca^2+^] that are associated with protein synthesis and expressed, at least in part, as a long-lasting increase in AMPAR activation that can be mediated by both presynaptic and postsynaptic alterations (Malenka and Bear, 2004). Induction of the long-lasting antidepressant-like effects of IGFI was blocked by the AMPA/kainate receptor antagonist NBQX and expressed as persistently enhanced AMPAergic transmission, consistent with our observation of increased GluR1 cell surface expression. This is also consistent with postsynaptic potentiation as measured by input/output analysis, with no effect on paired-pulse facilitation, suggesting no change in presynaptic function. Previous work in cell cultures suggests that IGFI can modulate Ca^2+^ influx (Kanzaki et al., 1999; Espinosa et al., 2004), a finding that remains to be tested for synaptically evoked Ca^2+^ influx in hippocampus and MPFC.

The facilitation of activity-dependent synaptic plasticity that can far outlast the presence of IGFI itself may underlie both antidepressant-like and nootropic effects of IGFI. Both conventional and rapid-acting antidepressants facilitate synaptic plasticity, in part by activating growth factor signaling (Brunoni et al., 2008; Duman and Aghajanian, 2012). In addition, growth factor activity has also been shown to facilitate learning and memory (Kopec and Carew, 2013). This suggests that antidepressant and nootropic effects of IGFI share a common mechanism.

IGFI-mediated synaptic plasticity differs from both conventional aminergic and novel NMDAR transmission-modulating antidepressants. IGFI potentiates synaptic transmission at CA1 Schaffer collateral-CA1 glutamatergic synapses and facilitates the induction of LTP-like synaptic plasticity through an IGFIR-dependent, but NMDAR-independent, manner. While IGFI is able to bypass NMDAR activation and potentiate glutamatergic synaptic transmission directly, IGFI still requires ongoing protein synthesis for its long-term effects. Since we found that IGFI-mediated potentiation, like NMDAR-LTP, is associated with enhanced AMPAR activation, these two signaling pathways may converge on a final common pathway to persistently enhance synaptic strength. In contrast to conventional antidepressants (electroconvulsive shock or chronic fluoxetine treatment), IGFI facilitates both LTP and basal synaptic transmission, whereas these conventional antidepressants inhibit the induction of LTP and facilitate postsynaptic basal transmission (Stewart and Reid, 2000).

The present study shows that IGFI exerts its antidepressant-like effects through a unique, NMDAR-independent mechanism, making the IGFI receptor complex a viable target for the development of novel compounds for the treatment of depression.

## Statement of Interest

None.
